# Tracking COVID-19 Infections Using Survey Data on Rapid At-Home Tests

**DOI:** 10.1001/jamanetworkopen.2024.35442

**Published:** 2024-09-30

**Authors:** Mauricio Santillana, Ata A. Uslu, Tamanna Urmi, Alexi Quintana-Mathe, James N. Druckman, Katherine Ognyanova, Matthew Baum, Roy H. Perlis, David Lazer

**Affiliations:** 1 Machine Intelligence Group for the Betterment of Health and the Environment, Northeastern University, Boston, Massachusetts; 2 Department of Epidemiology, Harvard T.H. Chan School of Public Health, Cambridge, Massachusetts; 3 Network Science Institute, Northeastern University, Boston, Massachusetts; 4 Department of Political Science, University of Rochester, Rochester, New York; 5 School of Communication and Information, Rutgers University, New Brunswick, New York; 6 Department of Government, John F. Kennedy School of Government, Harvard University, Cambridge, Massachusetts; 7 Department of Psychiatry, Massachusetts General Hospital and Harvard Medical School, Boston; 8 Editor, *JAMA Network Open*; 9 Department of Political Science, Northeastern University, Boston, Massachusetts; 10 Khoury College of Computer Sciences, Northeastern University, Boston, Massachusetts; 11 Institute for Quantitative Social Science, Harvard University, Cambridge, Massachusetts

## Abstract

**Importance:**

Identifying and tracking new infections during an emerging pandemic is crucial to design and deploy interventions to protect populations and mitigate the pandemic’s effects, yet it remains a challenging task.

**Objective:**

To characterize the ability of nonprobability online surveys to longitudinally estimate the number of COVID-19 infections in the population both in the presence and absence of institutionalized testing.

**Design, Setting, and Participants:**

Internet-based online nonprobability surveys were conducted among residents aged 18 years or older across 50 US states and the District of Columbia, using the PureSpectrum survey vendor, approximately every 6 weeks between June 1, 2020, and January 31, 2023, for a multiuniversity consortium—the COVID States Project. Surveys collected information on COVID-19 infections with representative state-level quotas applied to balance age, sex, race and ethnicity, and geographic distribution.

**Main Outcomes and Measures:**

The main outcomes were (1) survey-weighted estimates of new monthly confirmed COVID-19 cases in the US from January 2020 to January 2023 and (2) estimates of uncounted test-confirmed cases from February 1, 2022, to January 1, 2023. These estimates were compared with institutionally reported COVID-19 infections collected by Johns Hopkins University and wastewater viral concentrations for SARS-CoV-2 from Biobot Analytics.

**Results:**

The survey spanned 17 waves deployed from June 1, 2020, to January 31, 2023, with a total of 408 515 responses from 306 799 respondents (mean [SD] age, 42.8 [13.0] years; 202 416 women [66.0%]). Overall, 64 946 respondents (15.9%) self-reported a test-confirmed COVID-19 infection. National survey-weighted test-confirmed COVID-19 estimates were strongly correlated with institutionally reported COVID-19 infections (Pearson correlation, *r* = 0.96; *P* < .001) from April 2020 to January 2022 (50-state correlation mean [SD] value, *r* = 0.88 [0.07]). This was before the government-led mass distribution of at-home rapid tests. After January 2022, correlation was diminished and no longer statistically significant (*r* = 0.55; *P* = .08; 50-state correlation mean [SD] value, *r* = 0.48 [0.23]). In contrast, survey COVID-19 estimates correlated highly with SARS-CoV-2 viral concentrations in wastewater both before (*r* = 0.92; *P* < .001) and after (*r* = 0.89; *P* < .001) January 2022. Institutionally reported COVID-19 cases correlated (*r* = 0.79; *P* < .001) with wastewater viral concentrations before January 2022, but poorly (*r* = 0.31; *P* = .35) after, suggesting that both survey and wastewater estimates may have better captured test-confirmed COVID-19 infections after January 2022. Consistent correlation patterns were observed at the state level. Based on national-level survey estimates, approximately 54 million COVID-19 cases were likely unaccounted for in official records between January 2022 and January 2023.

**Conclusions and Relevance:**

This study suggests that nonprobability survey data can be used to estimate the temporal evolution of test-confirmed infections during an emerging disease outbreak. Self-reporting tools may enable government and health care officials to implement accessible and affordable at-home testing for efficient infection monitoring in the future.

## Key Points

**Question:**

Can nonprobability survey data accurately track institutionally confirmed COVID-19 cases in the US and provide estimates of unaccounted infections when rapid at-home tests are popularized and institutionalized tests are discontinued?

**Findings:**

In this survey study conducted among 306 799 residents aged 18 years or older across 50 US states and the District of Columbia, the proportion of individuals reporting a positive COVID-19 infection in a longitudinal nonprobability survey closely tracked the institutionally reported proportions in the US (15.9% self-reported a test-confirmed COVID-19 infection), as well as nationally aggregated wastewater SARS-CoV-2 viral concentrations, from April 2020 to February 2022. Survey estimates suggest that a high number of confirmed infections may have been unaccounted for in official records starting in February 2022, when large-scale distribution of rapid at-home tests occurred; this finding was further confirmed by viral concentrations in wastewater.

**Meaning:**

This study suggests that nonprobability online surveys can serve as an effective complementary method to monitor infections during an emerging pandemic and provide an alternative for estimating infections in the absence of institutional testing when at-home tests are widely available.

## Teaser

This survey study characterizes the ability of data from nonprobability online surveys to longitudinally estimate the number of COVID-19 infections in the population both in the presence and in the absence of institutionalized testing.

## Introduction

Identifying and tracking new infections during the earlier and most intense phases of the COVID-19 pandemic were crucial for the design of mitigation strategies; however, this was extremely challenging due to the novel nature of the pathogen.^1,2^ The significant number of asymptomatic COVID-19 infections, the limited availability of resources to identify and treat infections across locations, and people’s lack of trust and willingness to seek medical attention were some of the most important challenges of estimating incidence numbers.^3,4,5,6^ Multiple approaches to characterize the incidence of COVID-19 in the population were deployed in the US as infections spread.^7^ These approaches included (1) clinical-based individual testing (via polymerase chain reaction [PCR] or rapid tests)^8^; (2) tracking the number of patients in hospital visits with COVID-19 symptoms, such as fever, cough, sore throat, and anosmia (referred to as *syndromic surveillance*)^9^; (3) continuous monitoring of the presence of antibodies against SARS-CoV-2 in the blood serum samples in a population (referred to as *antigen testing and serosurveillance*)^3,10^; and (4) measuring the amount of SARS-CoV-2 viral concentration in wastewater (WW) samples shed by infected individuals.^4,11,12^

Among all these approaches, widespread institutional individual testing was the most heavily relied-on indicator to assess the severity of local outbreaks, allocate resources, and deploy or lift nonpharmaceutical mitigation interventions. Throughout the pandemic, however, testing availability and reporting were inconsistent in the US.^13^ For example, the COVID-19 tests—designed by the US Centers for Disease Control and Prevention—were recalled due to a faulty reagent^14^ during the earlier months of 2020, heterogenous state policies regarding access to free institutional testing led to inconsistencies in interpreting case count data,^15^ and the massive government-led distribution of rapid at-home tests starting in January 2022 without a concurrent deployment of a centralized infection reporting system meant that there was low coverage of testing.

Here, we study the ability of data collected from large US-based nonprobability surveys—the COVID States Project (CSP)—to estimate the number of COVID-19 infections from January 2020 to January 2023 at national and state levels. Multiple studies have investigated how surveys can be used to monitor infections, people’s behaviors, and trust in vaccines during specific periods and in particular geographical locations during the COVID-19 pandemic.^16,17^ In this study, we further sought to assess the extent to which carefully analyzed survey data could have been used to monitor the number of COVID-19 infections continuously and longitudinally at national and state levels during the first 3 years of the pandemic in the US.

## Methods

### Study Design

We used data collected by an ongoing large-scale internet-based nonprobability survey conducted by an academic consortium approximately every 6 weeks from June 1, 2020, to January 31, 2023, inclusive of all 50 states and the District of Columbia. Survey participants were individuals aged 18 years or older who resided in the US. Before agreeing to participate in the survey, respondents were not aware that the survey would include questions related to the COVID-19 pandemic, to minimize selection bias. The survey used national and state-level representative quotas for sex, age, and race and ethnicity (Asian, Black, Hispanic, White, and another race [no specific races or ethnicities were specified for “another race”]) to represent the US population in the most recent census data. Participants were recruited using PureSpectrum, an online survey panel aggregator, and they provided informed consent online before survey access. The study protocol was reviewed and approved by the institutional review board of Harvard University as exempt as only deidentified data were used and no participant contact was required. This study followed the American Association for Public Opinion Research (AAPOR) reporting guideline.

From the fifth survey wave (June 2020) onward, the surveys asked 2 questions to identify the COVID-19 test frequency of participants, positive test results, and the month when they experienced symptoms. The precise wording of the questions can be found in the eMethods in Supplement 1.

### Measures

All respondents were asked if they had been tested for COVID-19 in the past (not distinguishing between PCR test or antigen test in some waves), and those who indicated a positive test result were asked when they experienced symptoms. To estimate the number of infections happening in each month, we aggregated the number of respondents who indicated having a positive test result and were sick in each month, using only the immediately subsequent survey wave after each individual’s infection to minimize potential participants’ recall errors. Approximately 16% of respondents participated in multiple survey waves, and if they reported multiple infections in different months, we included their health status in each month that they reported an infection. Sensitivity analyses were conducted to test whether including more than 1 infection per respondent would yield different results compared with including at most only 1 (randomly selected) infection per respondent. The aggregated responses were demographically reweighted to represent the most recent US Census and normalized by the sample size to estimate the proportion of infected individuals at the national and state levels. The sample sizes and percentages of respondents who were sick in each month at the national level are shown in eTable 3 in Supplement 1. Institutionally confirmed COVID-19 infections were obtained from state and local governments and health departments by the Coronavirus Resource Center of Johns Hopkins University (JHU) and compiled by the *New York Times*. Finally, as an additional and independent measure of COVID-19 prevalence in the US, we used monthly aggregated WW SARS-CoV-2 viral concentrations from Biobot Analytics.^18^

### Statistical Analysis

We conducted our statistical analyses in 2 different and nonoverlapping time periods within the first 3 years of the pandemic in the US, selected a priori. The first period was from April 1, 2020, to January 31, 2022, a time when institutional efforts to test individuals were most active, according to the number of daily PCR COVID-19 tests conducted (eFigure 10 in Supplement 1). The second period was from February 1, 2022, to January 1, 2023, a time when rapid at-home tests were massively distributed to the general public by the federal government. During this period, there was not a centralized system to record the rapid test outcomes, and an overall decrease in governmental resources allocated to monitor COVID-19 infections gradually occurred, culminating with the federal public health emergency for COVID-19 expiring in May 2023.

#### Correlation Analysis

We calculated pairwise correlation coefficients between the proportion of infected individuals as inferred by survey data (referred to as *CSP*) and the institutional numbers reported in the JHU COVID-19 dashboard for the 2 time periods at the national and state levels. We also calculated pairwise correlations between SARS-CoV-2 viral load in WW and both the CSP estimates and the JHU reported infections during the 2 distinct time periods. This was done at the national and state levels.

#### Survey Mean Estimates and SEs

We measured the distance between the official numbers and our survey estimates as multiples of the survey-based SEs (SD of the mean) of our estimates. We report both these standardized differences and the 95% CIs in the Figure for the national level.

**Figure.**
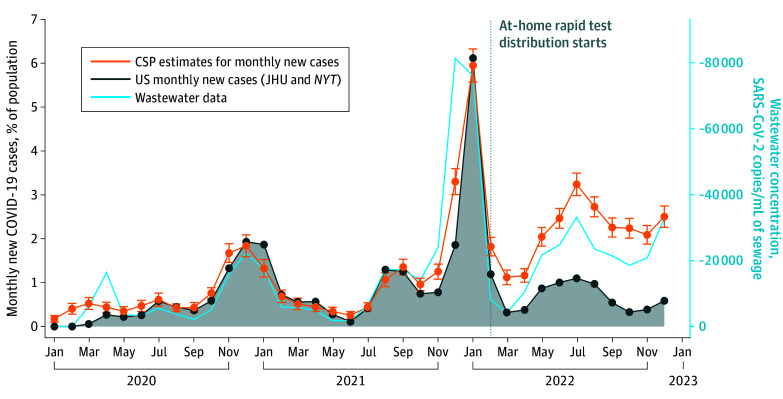
National Confirmed COVID-19 Infections, as Captured by Institutional and Survey Data, and Viral Concentrations of SARS-CoV-2 in Wastewater The percentage of respondents in our survey who reported having a confirmed COVID-19 infection in each month is shown in orange (CSP), the institutionally reported percentage of individuals infected in each month as monitored by Johns Hopkins University (JHU) is shown in dark blue, and the wastewater viral concentration of SARS-CoV-2 is shown in light blue. A vertical dotted line shows the time when at-home rapid tests were widely available in February 2022. *NYT* indicates the *New York Times*.

#### Excess Infection Estimates

We estimated the number of infections that were likely unaccounted for by institutional surveillance systems starting in February 2022 using 2 approaches. The first approach involved calculating the cumulative infections estimated from survey data from February 1, 2022, until January 1, 2023. This was achieved by multiplying the percentage of weight-corrected self-reported infected participants in each month by the total population of the US, and then by adding all these estimates across time. We then simply subtracted the cumulative number of infections reported by JHU from the survey’s cumulative estimates. A second approach involved calibrating a linear regression model to map CSP’s estimated incidence of COVID-19 infection onto JHU’s reported COVID-19 incidence from April 2020 to January 2022, to identify the association between the 2 models in the first 2 years when they closely tracked each other. We then used this model to estimate confirmed infections after February 2022, with the assumption that we had access only to CSP information. We computed cumulative values for the estimated JHU-reported incidence that would have been observed in the absence of any disruption (intervention) to the institutional COVID-19 surveillance, and then we compared these estimates with the cumulative cases calculated from JHU data. This method is detailed by De Salazar et al^19^ in the context of COVID-19 vaccine effectiveness and is frequently referred to as interrupted time-series analysis.^19,20^

## Results

The survey spanned 17 waves deployed from June 2020 to January 2023, with a total of 408 515 responses from 306 799 respondents (mean [SD] age, 42.8 [13.0] years; 202 416 respondents [66.0%] identified as women, and 104 383 respondents [34.0%] identified as men). A total of 16 715 respondents (5.4%) identified as Asian, 33 234 (10.8%) as Black, 24 938 (8.1%) as Hispanic, 219 448 (71.5%) as White, and 12 464 (4.1%) as another race. Overall, 64 946 respondents (15.9%) self-reported a test-confirmed COVID-19 infection. In aggregate and at the national level, COVID-19 case counts inferred from CSP surveys were highly correlated with JHU reports from April 2020 to January 2022 (Pearson *r* = 0.96; *P* < .001), as seen in the Figure and Table, with a mean (SD) state-level Pearson correlation coefficient *r* = 0.88 (0.07). After February 2022, soon after at-home rapid tests were massively distributed by the federal government, and up to January 2023, the Pearson correlation between CSP and JHU case counts decreased to *r* = 0.55 (*P* = .08) with a mean (SD) state-level Pearson correlation coefficient *r* = 0.48 (0.23). Sensitivity analysis shown in eFigure 11 in Supplement 1 yielded very similar temporal infection curves when including repeat participants only (randomly) once in our study. In addition, the Table shows that the concentrations of SARS-CoV-2 in WW data closely correlate with CSP surveys’ case counts both between April 2020 and January 2022 (*r* = 0.92; *P* < .001) and between February 2022 and January 2023 (*r* = 0.89; *P* < .001). Although SARS-CoV-2 in WW correlates (*r* = 0.79; *P* < .001) with JHU case counts before January 2022, this correlation decreased to *r* = 0.31 (*P* = .35) between February 2022 and January 2023. Consistent correlation patterns were observed at the state-level and are reported in eTables 1 to 5 and eFigures 4 and 9 in Supplement 1.

**Table.** National-Level Pairwise Pearson Correlations Between Survey Test-Confirmed Infection Estimates, Institutionally Reported COVID-19, and WW SARS-CoV-2 Viral Concentrations

Data source	April 2020-January 2022 (pre–rapid test period)		February 2022-January 2023 (rapid test period)		April 2020-January 2023 (full-time period)	
	Pearson correlation coefficient *r*	*P* value	Pearson correlation coefficient *r*	*P* value	Pearson correlation coefficient *r*	*P* value
CSP-JHU	0.96	<.001	0.55	.08	0.78	<.001
CSP-WW	0.92	<.001	0.89	< .001	0.87	<.001
JHU-WW	0.79	<.001	0.31	.35	0.74	<.001

Abbreviations: CSP, survey test-confirmed infections estimates; JHU, Johns Hopkins University; WW, wastewater.

Using the first approach to calculate cumulative infections from February 1, 2022, to January 1, 2023 (after at-home tests distribution), at the national level, our survey estimates suggest that approximately 79 million (95% CI, 71 million to 86 million) confirmed cases may have occurred compared with 25 million reported in the JHU data. This estimate indicates that 54 million cases, more than twice as many as those reported, were likely unaccounted for in institutional surveillance. At the state level, the number of potentially unaccounted cases varies between 59 000 in Wyoming and 6.3 million in California. Our second (interrupted time-series) approach, the linear regression (JHU = 0.96 × CSP – 0.1) calibrated during April 2020 to January 2022 nationally, yielded consistent results, and it estimated that the cumulative number of positive cases from February 1, 2022, to January 1, 2023, was 73 million (95% CI, 65 million to 81 million) at the national level. State-level results are consistent and shown in eTable 4 and eFigure 3 in Supplement 1.

For 22 months, from April 2020 (when reliable institutionalized testing in the US accelerated) until February 2022, case counts in the official data fell under within 2 to 3 SEs away from our estimates, except for the months of January, November, and December 2021. However, from February 2022 onward (when the distribution of rapid at-home tests started), the distance between survey estimates and the officially reported cases started to diverge significantly, ranging from 6 SEs in February 2022 to 16 SEs during the peak of July 2022 (eFigure 1 in Supplement 1).

## Discussion

Our results support the hypothesis that nonprobability surveys serve as a reliable and complementary method to monitor the proportion of test-confirmed infections in real time during a public health crisis. Specifically, by analyzing data from nonprobability surveys deployed approximately every 6 weeks during the first 2 years of the COVID-19 pandemic in the US, we found that the COVID-19 infections inferred from survey data closely tracked institutionally reported infections when institutional testing was at its best in the US. When institutional efforts to monitor COVID-19 infections diminished and rapid at-home tests were made widely accessible—with no centralized system to collect at-home test results—survey data suggested that a high proportion of test-confirmed infections were unaccounted for in institutional reports. When comparing with COVID-19 activity estimates obtained from SARS-CoV-2 concentrations in WW data, we found high consistency with the COVID-19 trends observed in surveys throughout this study.

The alignment of the 3 COVID-19 activity estimates—JHU, CSP, and WW—before January 2022 suggests that these surveillance systems were consistent and compatible with one another before the mass distribution of at-home tests. After February 2022, the consistency between CSP and WW COVID-19 activity, and the pronounced discordance between these 2 sources and JHU cases, suggest that both (1) CSP and WW data may have continued properly capturing COVID-19 infections trends and (2) the introduction of at-home rapid tests and the discontinuation of institutional testing disrupted institutional efforts (JHU) to track COVID-19 trends. Similar alignment between the 3 data sources was observed before January 2022 at the state level. There also were clear discrepancies between both CSP and WW data and JHU data after February 2022 at the state level, as shown in eFigures 2, 4, 5, and 6 in Supplement 1. Neither political affiliation, population size, nor health care spending per capita can explain the number of unreported infections per 100 000 inhabitants across states (eTable 5 in Supplement 1).

Although there have been multiple attempts to monitor or estimate the number of confirmed COVID-19 cases using alternative internet-based data sources, such as digital internet traces (eg, general population’s internet search queries and clinicians’ searches, among others^21^), human mobility data from smartphones,^22,23,24^ self-test reporting systems,^25^ and surveys such as ours,^26^ this study presents one of the most comprehensive assessments of the quality of COVID-19 activity estimates using nonprobability surveys, at the national and state levels, for the first 3 years of the pandemic.

Other attempts to track COVID-19 cases have used cross-sectional (or limited-period) surveys starting in the early stages of the pandemic. Most attempts concluded that only a small fraction of COVID-19 cases were captured by institutional testing, consistent with our findings after February 2022—and perhaps also during the early weeks of the pandemic.^5^ For example, a study by Gallup^27^ suggested that the number of COVID-19 infections on April 3, 2020, would be 2.5 times more than what the official numbers had suggested at that time if more people had undergone official testing. Another survey-based study conducted by Qasmieh et al^28^ fielded between March 14 and 16, 2022, asked 1030 adult residents of New York City about COVID-19 testing and related outcomes from January 2022 onward. They applied representative survey weights as in our method to estimate the number of infections in New York City. They estimated that 1.8 million adults (95% CI, 1.6 million to 2.1 million adults) had a COVID-19 infection from January 1 to March 16, 2022, compared with 1.1 million cases that our survey numbers suggest for the same period in New York state.

In another online survey-based (N = 97 707) study by Rader et al,^29^ researchers estimated that “2.6 million cases (95% CI, 1 874 549-3 853 341) were diagnosed by at-home tests and not included in the official case count” over the period from March 20 to May 21, 2022. Our surveys’ lower temporal resolution does not allow for a direct comparison with the daily estimates shown in the study by Rader et al.^29^ However, when scaling our monthly estimates (March estimate × 1/3 + April estimate + May estimate × 2/3), we estimate that approximately 6 million infections nationally were not included in reported case counts in the same period. These estimates, while both imperfect, confirm that millions of test-confirmed infections were missed in this overlapping time period.

In another survey-based study, Qasmieh et al^30^ estimated the cumulative incidence of COVID-19 cases during the preceding 14-day period (April 23 to May 8) to be 31 times the official case count: 1.5 million (95% CI, 1.3 million to 1.8 million) vs 50 000. In comparison, our estimates for April to May 2022 suggest that there were 1.2 million cases in New York state, much closer to the estimates in the study by Qasmieh et al.^30^ Government-led efforts aimed at centralizing information about individual at-home test results include a National Institutes of Health initiative tasked with developing a self-test reporting standard.

We identified large discrepancies in COVID-19 estimates among all 3 data sources—CSP, JHU, and WW—before May 2020. Both CSP and WW data showed significantly higher estimates of COVID-19 activity than those reported by JHU. Although testing was very sparse and inconsistent during this period, our estimates aligned with other attempts, for example,^21^ that have used statistical corrections and multiple complementary data streams to estimate the total number of COVID-19 infections before April 2020. Specifically, Lu et al^5^ found the cumulative number of suspected (symptomatic, either test confirmed or not) infections as of April 4, 2020, to be as many as 2.3 million to 4.8 million cases, or approximately 25 times the number of institutionally reported cases in the US. Our estimates do not point to such high numbers because, by design, our goal was to track only test-confirmed infections.

Because our surveys were not specifically designed to attract the participation of individuals with COVID-19 symptoms or particularly interested in COVID-19 more broadly, our infection rate estimates should be less biased than those obtained from COVID-19–specific surveys, such as the “Facebook-CMU” (Carnegie Mellon University) survey or “Outbreaks Near Me.”^31,32^ It has been documented that people who are experiencing symptoms are more motivated to report their experience in surveys,^33,34^ and thus incidence rates tend to be inflated in disease-specific surveys because fewer healthy individuals participate in them.

One strength of survey-based infection cases surveillance is that it allows for the multivariate collection of disease activity information in parallel with other sociodemographic variables. Institutional data collection, in contrast, rarely allows for access to demographic details of those reported to be infected and thus precludes examining subgroup infection rates. Future studies of the survey data should closely analyze infection trends in different sociodemographic groups.

In future public health crises, survey-based approaches to monitor confirmed infections should be deployed in conjunction with either widely available institutional testing or diagnostic at-home tests. In the absence of that, no criterion standard will exist to assess the historical validity of survey-based approaches, and, thus, their robustness and generalizability may be limited.

### Limitations

Our study has multiple limitations. The first is potential participants’ recall error. We used the answers from the most contemporaneous wave to estimate the number of infections in a month to mitigate recall bias. Another potential limitation might be entry errors in low-frequency responses. An expected low-frequency response in our study was whether the respondent was sick or not at the very beginning of the pandemic.^35^ Entry errors for low-frequency responses in an approximately 20 000-respondent wave (considered large) could have inflated our test-confirmed infection estimates in the first 3 months of the pandemic.

Although we have robust statistical power nationally and within large states as shown in eTables 2, 4, and 5 and eFigures 7 and 8 in Supplement 1, our state-level analyses are far less precise, especially for states where we had smaller sample sizes. For many, a probability sampling approach to estimate unreported infections would be preferable because such approaches are often deemed to be more representative of the population being studied.^36,37^ More work is needed to compare nonprobability and probability surveys in arriving at disease surveillance estimates. The main challenge for probability samples is the recent unit nonresponse bias that seems to be associated with the underrepresentation of particular groups of people.^38^ More importantly, probability samples are extremely expensive and logistically difficult. An advantage of our approach is that the logistics and lower price enable both spatial and temporal coverage, which are vital for careful and consistent disease surveillance.

## Conclusions

Our study supports the potential for applying surveys to complement government-led disease surveillance in future public health crises, despite some limitations that may be addressable in future deployments. Self-reporting tools may enable government and health care officials to implement accessible and affordable at-home testing for efficient infection monitoring in the future.

## Notes

Supplement 1.
eMethods.
**eFigure 1.** Top panel: The percent of respondents in our survey (CSP) who reported having a confirmed COVID-19 infection in each month is shown in red, the institutionally reported percent of individuals infected in each month as monitored by JHU is shown in black, and the wastewater viral concentration of SARS-CoV-2 is shown in blue. A vertical green dashed line shows the time when at-home rapid test were widely delivered in February 2022. Bottom panel: differences between CSP and JHU new monthly infections as multiples of the standard error (of means) of the CSP estimates. **eTable 1.** Average Pearson correlation for all US states between survey test-confirmed infections estimates (CSP), Institutionally reported COVID-19 (JHU), and Wastewater SARS-CoV-2 viral concentrations (WW) in two time periods **eFigure 2.** COVID-19 case estimates from CSP data (with confidence intervals, in red), concentrations of SARS-CoV-2 in wastewater (blue), and institutional confirmed cases from JHU (black) for all US states **eFigure 3.** Scatter plots of JHU COVID-19 infections vs CSP infections from April 2020 to January 2022 was used for training (blue) and from February 2022 to January 2023 (red) **eFigure 4.** Scatter plots of JHU COVID-19 infections vs CSP infections from April 2020 to January 2022 was used for training (blue) and from February 2022 to January 2023 (red) at the State-level **eFigure 5.** COVID-19 case estimates from Covid States Project data (with confidence interval) and official data source for all states **eFigure 6.** Interrupted time-series approach: COVID-19 case estimates from JHU (black) and predictions using interrupted linear regression relating official source and Covid States Project for all states during April 2020 and January 2022 **eFigure 7.** Data availability from Biobot’s wastewater sequencing per month in each state **eFigure 8.** Data availability from COVID states project survey per month in each state **eTable 2.** State-level pairwise Pearson correlation and p-values between survey test-confirmed infections estimates (CSP), Institutionally reported COVID-19 (JHU), and Wastewater SARS-CoV-2 viral concentrations (WW) in three time periods **eTable 3.** Monthly observed COVID-19 cases estimated from survey data for the multiple survey deployments **eTable 4.** The differences in number of cases recorded during the period after rapid tests were deployed on ground (Feb’22 to Dec’22) between Official data source (New York Times) and Covid States survey and prediction obtained by training a linear regression using Covid states **eTable 5.** Number of state-level unreported infection per 100,000 individuals as calculated from survey data **eFigure 9.** Case estimation performed by BioBot using viral concentration in wastewater **eFigure 10.** Daily COVID-19 tests administered in the United States per thousand people **eFigure 11.** Sensitivity analysis of infection curves obtained by only including repeat respondents only once (chosen randomly) in our longitudinal analysis

Supplement 2.
Data Sharing Statement
