# Supplementary material for: Tracking COVID-19 Infections Using Survey Data on Rapid At-Home Tests
Source: JAMA Netw Open. 2024 Sep 30;7(9):e2435442. doi: 10.1001/jamanetworkopen.2024.35442 (PMC11443354; doi:10.1001/jamanetworkopen.2024.35442)
Supplement: Supplement 1. — eMethods. eFigure 1. Top panel: The percent of respondents in our survey (CSP) who reported having a confirmed COVID-19 infection in each month is shown in red, the institutionally reported percent of individuals infected in each month as monitored by JHU is shown in black, and the wastewater viral concentration of SARS-CoV-2 is shown in blue. A vertical green dashed line shows the time when at-home rapid test were widely delivered in February 2022. Bottom panel: differences between CSP and JHU new monthly infections as multiples of the standard error (of means) of the CSP estimates. eTable 1. Average Pearson correlation for all US states between survey test-confirmed infections estimates (CSP), Institutionally reported COVID-19 (JHU), and Wastewater SARS-CoV-2 viral concentrations (WW) in two time periods eFigure 2. COVID-19 case estimates from CSP data (with confidence intervals, in red), concentrations of SARS-CoV-2 in wastewater (blue), and institutional confirmed cases from JHU (black) for all US states eFigure 3. Scatter plots of JHU COVID-19 infections vs CSP infections from April 2020 to January 2022 was used for training (blue) and from February 2022 to January 2023 (red) eFigure 4. Scatter plots of JHU COVID-19 infections vs CSP infections from April 2020 to January 2022 was used for training (blue) and from February 2022 to January 2023 (red) at the State-level eFigure 5. COVID-19 case estimates from Covid States Project data (with confidence interval) and official data source for all states eFigure 6. Interrupted time-series approach: COVID-19 case estimates from JHU (black) and predictions using interrupted linear regression relating official source and Covid States Project for all states during April 2020 and January 2022 eFigure 7. Data availability from Biobot’s wastewater sequencing per month in each state eFigure 8. Data availability from COVID states project survey per month in each state eTable 2. State-level pairwise Pearson correlation and p-va [file jamanetwopen-e2435442-s001.pdf]

Supplementary Online Content

Santillana M, Uslu AA, Urmi T, et al. Tracking COVID-19 infections using survey data on rapid at-home tests. *JAMA Netw Open.* 2024;7(9):e2435442.
doi:10.1001/jamanetworkopen.2024.35442

eMethods.
eFigure 1. Top panel: The percent of respondents in our survey (CSP) who reported having a confirmed COVID-19 infection in each month is shown in red, the institutionally reported percent of individuals infected in each month as monitored by JHU is shown in black, and the wastewater viral concentration of SARS-CoV-2 is shown in blue. A vertical green dashed line shows the time when at-home rapid test were widely delivered in February 2022. Bottom panel: differences between CSP and JHU new monthly infections as multiples of the standard error (of means) of the CSP estimates.
eTable 1. Average Pearson correlation for all US states between survey test-confirmed infections estimates (CSP), Institutionally reported COVID-19 (JHU), and Wastewater SARS-CoV-2 viral concentrations (WW) in two time periods
eFigure 2. COVID-19 case estimates from CSP data (with confidence intervals, in red), concentrations of SARS-CoV-2 in wastewater (blue), and institutional confirmed cases from JHU (black) for all US states
eFigure 3. Scatter plots of JHU COVID-19 infections vs CSP infections from April 2020 to January 2022 was used for training (blue) and from February 2022 to January 2023 (red)
eFigure 4. Scatter plots of JHU COVID-19 infections vs CSP infections from April 2020 to January 2022 was used for training (blue) and from February 2022 to January 2023 (red) at the State-level
eFigure 5. COVID-19 case estimates from Covid States Project data (with confidence interval) and official data source for all states
eFigure 6. Interrupted time-series approach: COVID-19 case estimates from JHU (black) and predictions using interrupted linear regression relating official source and Covid States Project for all states during April 2020 and January 2022
eFigure 7. Data availability from Biobot's wastewater sequencing per month in each state
eFigure 8. Data availability from COVID states project survey per month in each state
eTable 2. State-level pairwise Pearson correlation and p-values between survey test-confirmed infections estimates (CSP), Institutionally reported COVID-19 (JHU), and Wastewater SARS-CoV-2 viral concentrations (WW) in three time periods
eTable 3. Monthly observed COVID-19 cases estimated from survey data for the multiple survey deployments
eTable 4. The differences in number of cases recorded during the period after rapid tests were deployed on ground (Feb'22 to Dec'22) between Official data source (New York Times) and Covid States survey and prediction obtained by training a linear regression using Covid states
eTable 5. Number of state-level unreported infection per 100,000 individuals as calculated from survey data
eFigure 9. Case estimation performed by BioBot using viral concentration in wastewater
eFigure 10. Daily COVID-19 tests administered in the United States per thousand people
eFigure 11. Sensitivity analysis of infection curves obtained by only including repeat respondents only once (chosen randomly) in our longitudinal analysis

This supplementary material has been provided by the authors to give readers additional information about their work.

© 2024 Santillana M et al. *JAMA Network Open.*

eMethods.

Precise wording of questions on survey about COVID-19 infections:

Question 1. Have you been tested for coronavirus (COVID-19)?

- Yes, and I tested positive for COVID-19 at least once
- Yes, and I tested negative for COVID-19 every time
- No, I wanted to but was not able to get a test
- No, I never tried to get tested

If answer is yes for Question 1, then:
Question 2. In which months of 2020 were you sick? (Please select all that apply)

- January 2020
- February 2020
- March 2020
- …
- December 2022

We highlight the fact that when we first conceived the set of COVID-19 infection questions in our survey, the biology behind potential COVID-19 re-infections was not clearly understood (and thus not fully expected) and rapid at-home tests were not available. Over time, both the availability of rapid tests became ubiquitous and the fact that people could be re-infected (more than once) became a reality. **We chose to maintain temporal consistency in our survey and did not change the phrasing of the COVID-19 infections questions**. When repeat infections occurred due to new variants, a new test should have been expected to be associated with the timing of a new infection. In short, in this study, we treat the month in which symptoms appeared as the month of a positive COVID-19 case, irrespective of the test status for those specific symptoms (given that the test status was only queried in terms of ever having tested positive).

Recruitment of repeat respondents

The recruitment process for repeat respondents could bias our study. We note, however, that repeat respondents were not aware that this was the same survey when they were recruited in a subsequent wave. In order to assess the effect of this potential bias, however, we obtained estimates of unreported infections by removing repeat participants (about 16% of the sample) completely. The result of this process, as highlighted later in this document (Supplementary materials) was that generally, our results remain consistent with all the statements throughout our study.

AAPOR guidelines

One of the canonical pieces of information provided in survey research in probability samples is the response rate, as detailed in the AAPOR guidelines. Yet, as an AAPOR report explains, “In non-probability samples, the denominator for the ratio may not be known, therefore it is not always possible to produce response rates as traditionally defined by AAPOR and other

© 2024 Santillana M et al. *JAMA Network Open.*

professional standards bodies"[1]. We now report two metrics to better inform our readers: 1) the percentage of people who passed our data quality screeners, and 2) the break-off rate or the percentage of people who started but did not complete the survey. Specifically, across all of our survey waves, between 5% and 31% have been disqualified for quality, on average the percent is 23%. Across all our survey waves, 11% to 25% of those who start have dropped out, the average being 17%.

[1] Reg Baker, J. Michael Brick, Nancy A. Bates, Mike Battaglia, Mick P. Couper, Jill A. Dever, Krista J. Gile, Roger Tourangeau, Summary Report of the AAPOR Task Force on Non-probability Sampling, *Journal of Survey Statistics and Methodology*, Volume 1, Issue 2, November 2013, Pages 90–143, https://doi.org/10.1093/jssam/smt008

NPS_TF_Report_Final_7_revised_FNL_6_22_13-2.pdf (aapor.org)

© 2024 Santillana M et al. *JAMA Network Open.*

Results

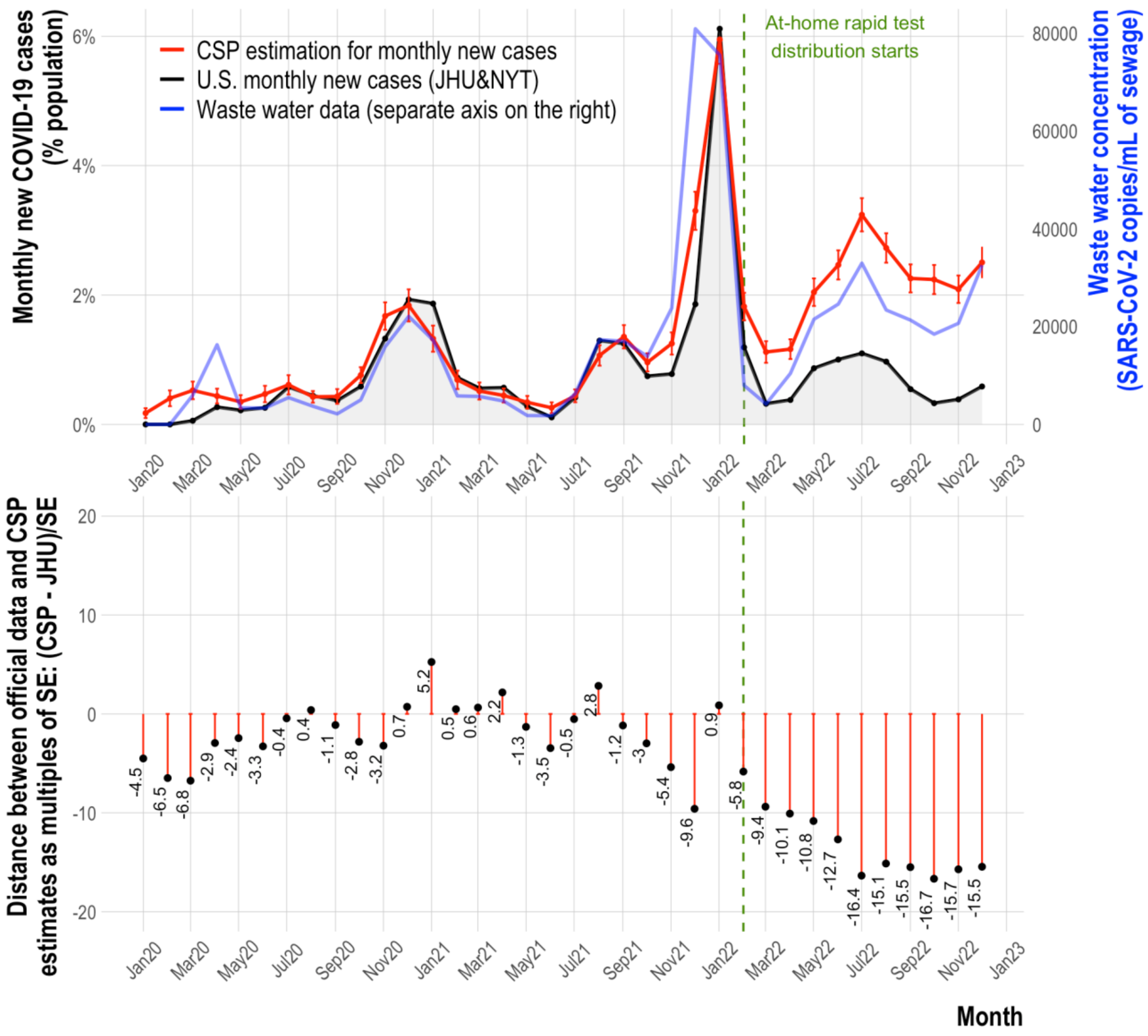

eFigure 1. Top panel: The percent of respondents in our survey (CSP) who reported having a confirmed COVID-19 infection in each month is shown in red, the institutionally reported percent of individuals infected in each month as monitored by JHU is shown in black, and the wastewater viral concentration of SARS-CoV-2 is shown in blue. A vertical green dashed line shows the time when at-home rapid test were widely delivered in February 2022. Bottom panel: differences between CSP and JHU new monthly infections as multiples of the standard error (of means) of the CSP estimates.

© 2024 Santillana M et al. *JAMA Network Open.*

eTable 1. Average Pearson correlation for all US states between survey test-confirmed infections estimates (CSP), Institutionally reported COVID-19 (JHU), and Wastewater SARS-CoV-2 viral concentrations (WW) in two time periods

Period	CSP-JHU	WW-CSP	JHU-WW
Apr 2020 – Jan 2022 (pre-rapid test period)	0.882 (SD = 0.073)	0.614 (SD = 0.374)	0.652 (SD = 0.290)
Feb 2022 – Jan 2023 (rapid test period)	0.48 (SD = 0.227)	0.470 (SD = 0.314)	0.146 (SD = 0.322)

© 2024 Santillana M et al. *JAMA Network Open.*

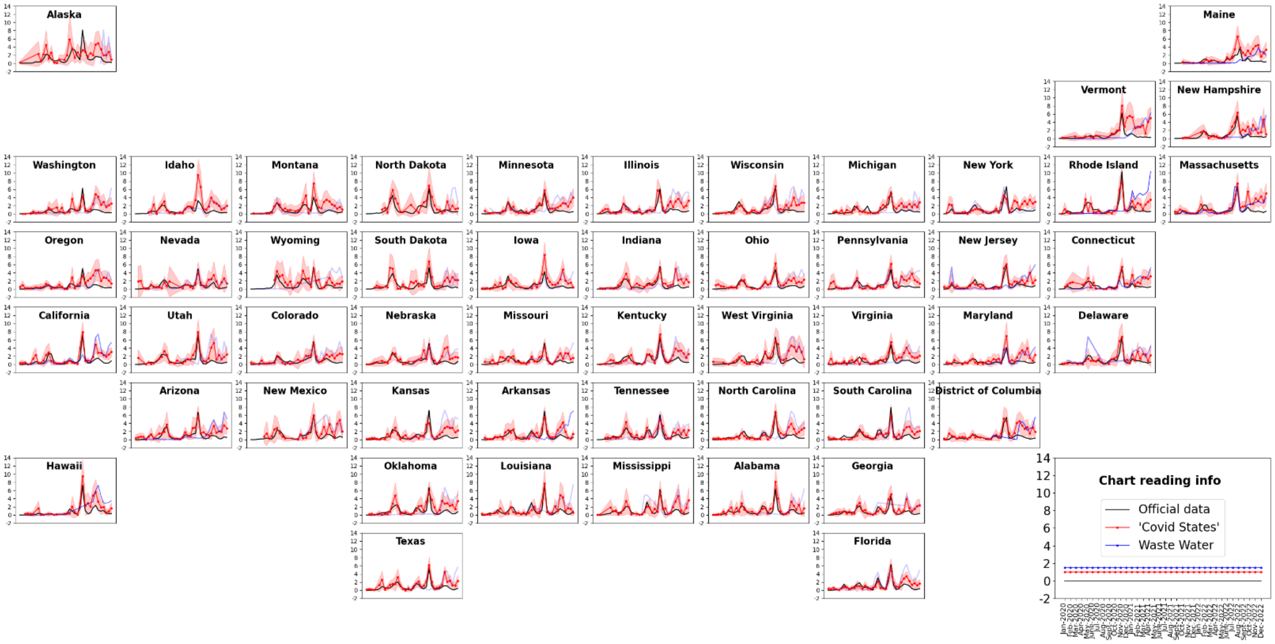

eFigure 2. COVID-19 case estimates from CSP data (with confidence intervals, in red), concentrations of SARS-CoV-2 in wastewater (blue), and institutional confirmed cases from JHU (black) for all US states. For Waste water concentrations, darker blue colors indicate the presence of higher numbers of observation stations and thus, more robust estimates.

© 2024 Santillana M et al. *JAMA Network Open.*

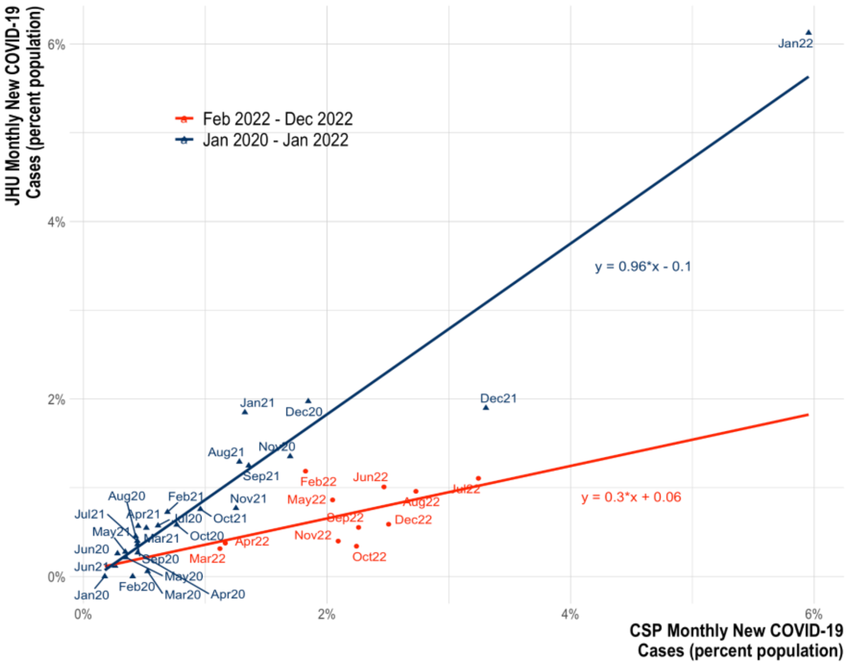

eFigure 3. Scatter plots of JHU COVID-19 infections vs CSP infections from April 2020 to January 2022 was used for training (blue) and from February 2022 to January 2023 (red). Individually fitted linear regressions for both time periods and their equations are included. Using the regression on the time period from April 2020 to January 2022 as our **interrupted time series** approach, we calculated the infections that would have been observed in the JHU data during the time period February 2022 to January 2023, had rapid at-home tests not been widely distributed and JHU surveillance had not been dramatically reduced.

© 2024 Santillana M et al. *JAMA Network Open.*

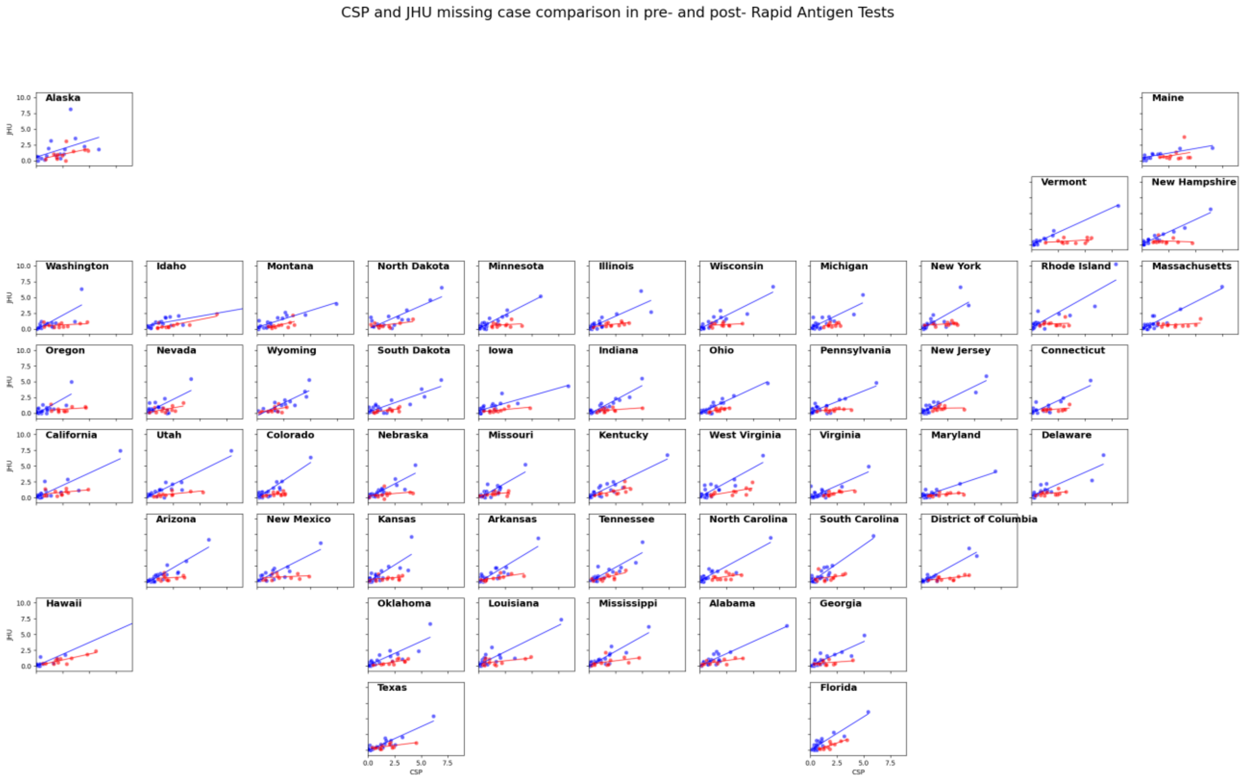

eFigure 4. Scatter plots of JHU COVID-19 infections vs CSP infections from April 2020 to January 2022 was used for training (blue) and from February 2022 to January 2023 (red) at the State-level (Similar to Figure S3). Individually fitted linear regressions for both time periods and their equations are included. Using the regression on the time period from April 2020 to January 2022 as our **interrupted time series** approach, we calculated the infections that would have been observed in the JHU data during the time period February 2022 to January 2023, had rapid at-home tests not been widely distributed and JHU surveillance had not been dramatically reduced.

© 2024 Santillana M et al. *JAMA Network Open.*

State-wise case estimation and data volume

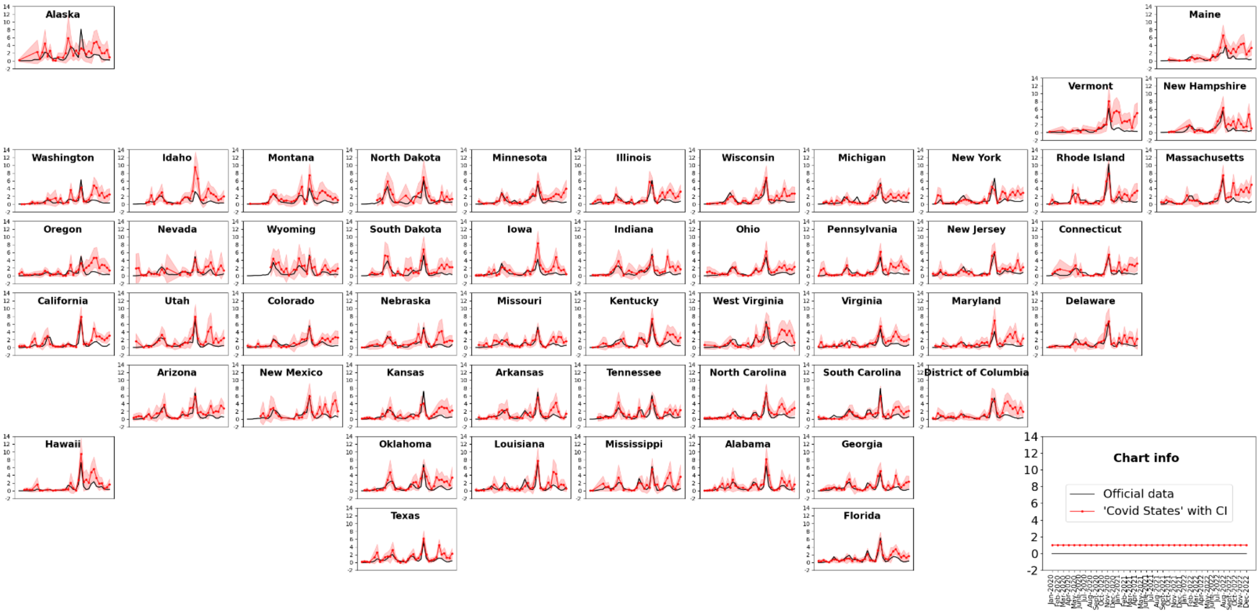

eFigure 5. COVID-19 case estimates from Covid States Project data (with confidence interval) and official data source for all states

© 2024 Santillana M et al. *JAMA Network Open.*

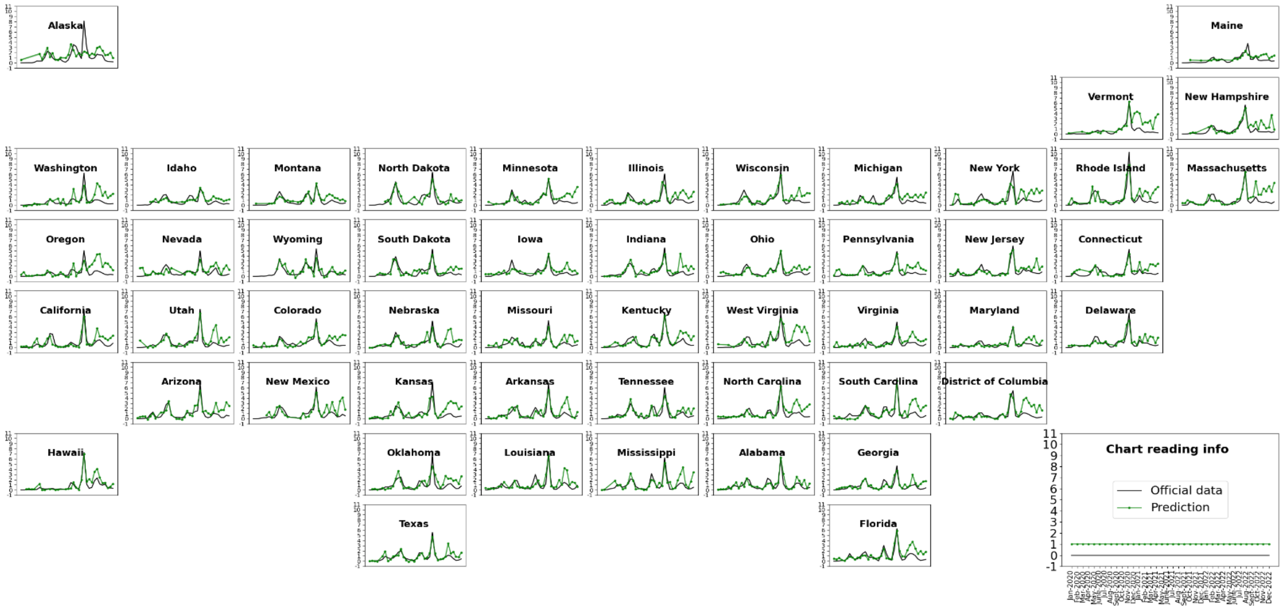

eFigure 6. Interrupted time-series approach: COVID-19 case estimates from JHU (black) and predictions using interrupted linear regression relating official source and Covid States Project for all states during April 2020 and January 2022.

© 2024 Santillana M et al. *JAMA Network Open.*

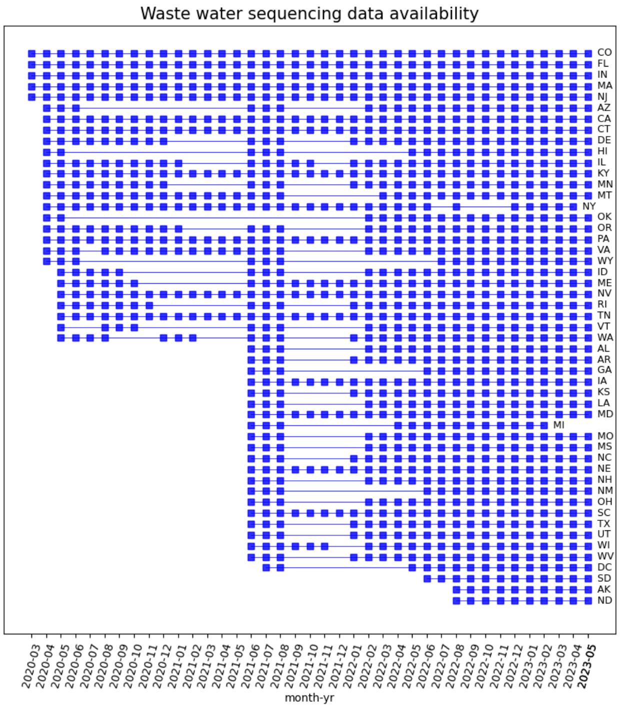

eFigure 7. Data availability from Biobot's wastewater sequencing per month in each state. Months with no data has been left blank and those with data has been marked with blue squares.

© 2024 Santillana M et al. *JAMA Network Open.*

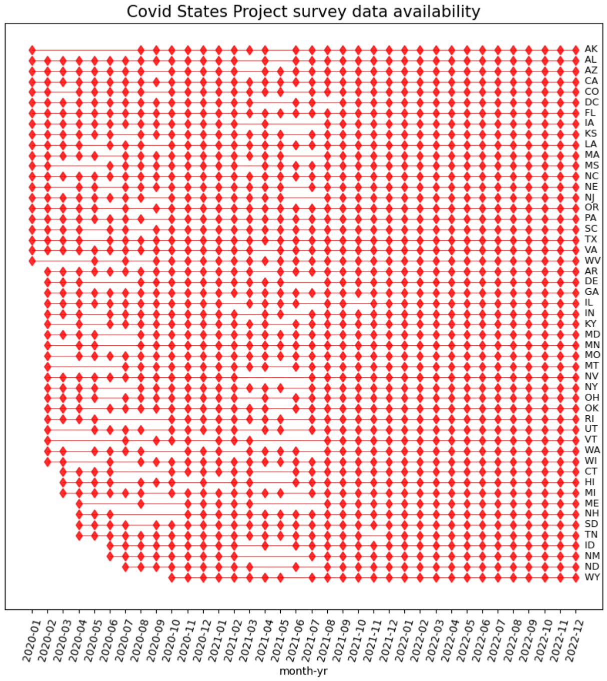

eFigure 8. Data availability from COVID states project survey per month in each state. Months with no data has been left blank and those with data has been marked with red circles.

© 2024 Santillana M et al. *JAMA Network Open.*

eTable 2. State-level pairwise Pearson correlation and p-values between survey test-confirmed infections estimates (CSP), Institutionally reported COVID-19 (JHU), and Wastewater SARS-CoV-2 viral concentrations (WW) in three time periods.

	state	CSP - JHU	WW - CSP	JHU - WW
**Apr 2020 – Jan 2023 (full data)**	CO	0.7236 (p = 2e-06)	0.7583 (p = 0.0)	0.3163 (p = 0.072877)
**Apr 2020 – Jan 2022 (pre-rapid test period)**	CO	0.9151 (p = 0.0)	0.8679 (p = 0.0)	0.7713 (p = 2.6e-05)
**Feb 2022 – Jan 2023 (rapid test period)**	CO	0.0624 (p = 0.855307)	0.6247 (p = 0.039877)	-0.0931 (p = 0.785523)
**Apr 2020 – Jan 2023 (full data)**	FL	0.7779 (p = 0.0)	0.781 (p = 0.0)	0.4276 (p = 0.013055)
**Apr 2020 – Jan 2022 (pre-rapid test period)**	FL	0.9332 (p = 0.0)	0.771 (p = 2.7e-05)	0.741 (p = 8e-05)
**Feb 2022 – Jan 2023 (rapid test period)**	FL	0.8204 (p = 0.001977)	0.8227 (p = 0.001874)	0.5378 (p = 0.087921)
**Apr 2020 – Jan 2023 (full data)**	IN	0.7062 (p = 4e-06)	0.5331 (p = 0.001403)	0.0528 (p = 0.770285)
**Apr 2020 – Jan 2022 (pre-rapid test period)**	IN	0.9233 (p = 0.0)	0.7829 (p = 1.7e-05)	0.811 (p = 5e-06)
**Feb 2022 – Jan 2023 (rapid test period)**	IN	0.473 (p = 0.141719)	0.6645 (p = 0.025714)	0.5626 (p = 0.071605)
**Apr 2020 – Jan 2023 (full data)**	MA	0.6321 (p = 7.9e-05)	0.852 (p = 0.0)	0.6843 (p = 1.1e-05)
**Apr 2020 – Jan 2022 (pre-rapid test period)**	MA	0.9425 (p = 0.0)	0.868 (p = 0.0)	0.8645 (p = 0.0)
**Feb 2022 – Jan 2023 (rapid test period)**	MA	0.3776 (p = 0.252319)	0.7323 (p = 0.010383)	-0.0626 (p = 0.854949)
**Apr 2020 – Jan 2023 (full data)**	NJ	0.805 (p = 0.0)	0.3883 (p = 0.025526)	0.0624 (p = 0.730301)
**Apr 2020 – Jan 2022 (pre-rapid test period)**	NJ	0.9414 (p = 0.0)	0.2781 (p = 0.21008)	0.2159 (p = 0.334449)
**Feb 2022 – Jan 2023 (rapid test period)**	NJ	0.0577 (p = 0.8662)	0.4139 (p = 0.205712)	0.042 (p = 0.902506)
**Apr 2020 – Jan 2023 (full data)**	CA	0.752 (p = 0.0)	0.5897 (p = 0.000305)	0.2327 (p = 0.192554)
**Apr 2020 – Jan 2022 (pre-rapid test period)**	CA	0.865 (p = 0.0)	0.7254 (p = 0.000133)	0.7878 (p = 1.3e-05)
**Feb 2022 – Jan 2023 (rapid test period)**	CA	0.414 (p = 0.205536)	0.8567 (p = 0.000757)	0.5687 (p = 0.067881)
**Apr 2020 – Jan 2023 (full data)**	CT	0.6849 (p = 1.1e-05)	0.5595 (p = 0.000712)	0.0747 (p = 0.679505)
**Apr 2020 – Jan 2022 (pre-rapid test period)**	CT	0.874 (p = 0.0)	0.5454 (p = 0.008662)	0.5765 (p = 0.004978)
**Feb 2022 – Jan 2023 (rapid test period)**	CT	0.4207 (p = 0.197546)	0.522 (p = 0.099571)	-0.0726 (p = 0.83195)
**Apr 2020 – Jan 2023 (full data)**	KY	0.7944 (p = 0.0)	0.7994 (p = 0.0)	0.4976 (p = 0.003215)
**Apr 2020 – Jan 2022 (pre-rapid test period)**	KY	0.9165 (p = 0.0)	0.9133 (p = 0.0)	0.8949 (p = 0.0)
**Feb 2022 – Jan 2023 (rapid test period)**	KY	0.6155 (p = 0.043819)	0.7627 (p = 0.006336)	0.3957 (p = 0.228368)
**Apr 2020 – Jan 2023 (full data)**	PA	0.715 (p = 3e-06)	0.7822 (p = 0.0)	0.3884 (p = 0.025489)
**Apr 2020 – Jan 2022 (pre-rapid test period)**	PA	0.954 (p = 0.0)	0.8659 (p = 0.0)	0.8317 (p = 2e-06)
**Feb 2022 – Jan 2023 (rapid test period)**	PA	0.502 (p = 0.115618)	0.4562 (p = 0.158407)	0.2932 (p = 0.381495)
**Apr 2020 – Jan 2023 (full data)**	NV	0.5962 (p = 0.000251)	0.8064 (p = 0.0)	0.7173 (p = 3e-06)
**Apr 2020 – Jan 2022 (pre-rapid test period)**	NV	0.8533 (p = 0.0)	0.8077 (p = 5e-06)	0.8529 (p = 0.0)
**Feb 2022 – Jan 2023 (rapid test period)**	NV	0.5533 (p = 0.077471)	0.8126 (p = 0.002367)	0.4995 (p = 0.117759)
**Apr 2020 – Jan 2023 (full data)**	TN	0.7889 (p = 0.0)	0.6269 (p = 9.5e-05)	0.5667 (p = 0.000585)
**Apr 2020 – Jan 2022 (pre-rapid test period)**	TN	0.8602 (p = 0.0)	0.7261 (p = 0.00013)	0.8706 (p = 0.0)
**Feb 2022 – Jan 2023 (rapid test period)**	TN	0.7364 (p = 0.009749)	0.4238 (p = 0.19395)	0.2652 (p = 0.430663)

© 2024 Santillana M et al. *JAMA Network Open.*

**Apr 2020 – Jan 2023 (full data)**	NY	0.5794 (p = 0.00041)	0.4821 (p = 0.004498)	0.6896 (p = 9e-06)
**Apr 2020 – Jan 2022 (pre-rapid test period)**	NY	0.8133 (p = 4e-06)	0.7915 (p = 1.1e-05)	0.6958 (p = 0.000323)
**Feb 2022 – Jan 2023 (rapid test period)**	NY	0.4346 (p = 0.181646)	Insufficient data	Insufficient data
**Apr 2020 – Jan 2023 (full data)**	IL	0.6639 (p = 2.5e-05)	0.4203 (p = 0.014888)	-0.0391 (p = 0.828884)
**Apr 2020 – Jan 2022 (pre-rapid test period)**	IL	0.8307 (p = 2e-06)	-0.0372 (p = 0.869481)	0.1542 (p = 0.493137)
**Feb 2022 – Jan 2023 (rapid test period)**	IL	0.6073 (p = 0.047527)	0.5006 (p = 0.116792)	0.3405 (p = 0.305485)
**Apr 2020 – Jan 2023 (full data)**	MT	0.7966 (p = 0.0)	0.1044 (p = 0.563299)	0.0601 (p = 0.739731)
**Apr 2020 – Jan 2022 (pre-rapid test period)**	MT	0.9095 (p = 0.0)	Insufficient data	Insufficient data
**Feb 2022 – Jan 2023 (rapid test period)**	MT	0.6307 (p = 0.037483)	-0.4499 (p = 0.165025)	-0.4599 (p = 0.154633)
**Apr 2020 – Jan 2023 (full data)**	VA	0.679 (p = 1.4e-05)	0.4774 (p = 0.004958)	-0.0232 (p = 0.897852)
**Apr 2020 – Jan 2022 (pre-rapid test period)**	VA	0.8535 (p = 0.0)	-0.3065 (p = 0.165369)	0.0564 (p = 0.803179)
**Feb 2022 – Jan 2023 (rapid test period)**	VA	0.7241 (p = 0.011743)	0.4751 (p = 0.139761)	0.1832 (p = 0.589743)
**Apr 2020 – Jan 2023 (full data)**	ME	0.5703 (p = 0.000531)	0.6131 (p = 0.000148)	0.0276 (p = 0.878888)
**Apr 2020 – Jan 2022 (pre-rapid test period)**	ME	Insufficient data	Insufficient data	Insufficient data
**Feb 2022 – Jan 2023 (rapid test period)**	ME	0.2653 (p = 0.430362)	0.2518 (p = 0.455069)	-0.4202 (p = 0.198149)
**Apr 2020 – Jan 2023 (full data)**	DE	0.7908 (p = 0.0)	0.1926 (p = 0.282968)	0.0551 (p = 0.760699)
**Apr 2020 – Jan 2022 (pre-rapid test period)**	DE	0.8957 (p = 0.0)	Insufficient data	Insufficient data
**Feb 2022 – Jan 2023 (rapid test period)**	DE	0.4068 (p = 0.214426)	0.4697 (p = 0.144952)	0.3762 (p = 0.254185)
**Apr 2020 – Jan 2023 (full data)**	MN	0.6939 (p = 8e-06)	0.4326 (p = 0.011913)	-0.1146 (p = 0.525347)
**Apr 2020 – Jan 2022 (pre-rapid test period)**	MN	0.9526 (p = 0.0)	Insufficient data	Insufficient data
**Feb 2022 – Jan 2023 (rapid test period)**	MN	0.2159 (p = 0.523648)	0.3322 (p = 0.318164)	-0.1257 (p = 0.71262)
**Apr 2020 – Jan 2023 (full data)**	OR	0.4321 (p = 0.012034)	0.6671 (p = 2.2e-05)	-0.0153 (p = 0.93258)
**Apr 2020 – Jan 2022 (pre-rapid test period)**	OR	0.7893 (p = 1.3e-05)	Insufficient data	Insufficient data
**Feb 2022 – Jan 2023 (rapid test period)**	OR	0.3145 (p = 0.346187)	0.4303 (p = 0.18647)	0.0012 (p = 0.997249)
**Apr 2020 – Jan 2023 (full data)**	RI	0.7828 (p = 0.0)	Insufficient data	Insufficient data
**Apr 2020 – Jan 2022 (pre-rapid test period)**	RI	0.9012 (p = 0.0)	Insufficient data	Insufficient data
**Feb 2022 – Jan 2023 (rapid test period)**	RI	0.1323 (p = 0.698122)	0.8303 (p = 0.001555)	-0.0747 (p = 0.827154)
**Apr 2020 – Jan 2023 (full data)**	WA	0.5164 (p = 0.002096)	Insufficient data	Insufficient data
**Apr 2020 – Jan 2022 (pre-rapid test period)**	WA	0.7744 (p = 2.3e-05)	Insufficient data	Insufficient data
**Feb 2022 – Jan 2023 (rapid test period)**	WA	0.2944 (p = 0.379483)	0.6755 (p = 0.022531)	-0.1275 (p = 0.708757)
**Apr 2020 – Jan 2023 (full data)**	ID	0.7438 (p = 1e-06)	Insufficient data	Insufficient data
**Apr 2020 – Jan 2022 (pre-rapid test period)**	ID	0.8119 (p = 4e-06)	Insufficient data	Insufficient data
**Feb 2022 – Jan 2023 (rapid test period)**	ID	0.8588 (p = 0.00071)	0.3319 (p = 0.318764)	-0.0347 (p = 0.919299)
**Apr 2020 – Jan 2023 (full data)**	IA	0.7188 (p = 2e-06)	Insufficient data	Insufficient data
**Apr 2020 – Jan 2022 (pre-rapid test period)**	IA	0.8597 (p = 0.0)	Insufficient data	Insufficient data
**Feb 2022 – Jan 2023 (rapid test period)**	IA	0.6457 (p = 0.031862)	0.329 (p = 0.323265)	0.0882 (p = 0.796618)
**Apr 2020 – Jan 2023 (full data)**	MD	0.7618 (p = 0.0)	Insufficient data	Insufficient data

© 2024 Santillana M et al. *JAMA Network Open.*

**Apr 2020 – Jan 2022 (pre-rapid test period)**	MD	0.9291 (p = 0.0)	Insufficient data	Insufficient data
**Feb 2022 – Jan 2023 (rapid test period)**	MD	0.3351 (p = 0.313778)	0.4576 (p = 0.156968)	-0.024 (p = 0.944097)
**Apr 2020 – Jan 2023 (full data)**	NE	0.6265 (p = 9.6e-05)	Insufficient data	Insufficient data
**Apr 2020 – Jan 2022 (pre-rapid test period)**	NE	0.8651 (p = 0.0)	Insufficient data	Insufficient data
**Feb 2022 – Jan 2023 (rapid test period)**	NE	0.3343 (p = 0.315053)	0.5225 (p = 0.099164)	0.0034 (p = 0.992116)
**Apr 2020 – Jan 2023 (full data)**	SC	0.7537 (p = 0.0)	Insufficient data	Insufficient data
**Apr 2020 – Jan 2022 (pre-rapid test period)**	SC	0.934 (p = 0.0)	Insufficient data	Insufficient data
**Feb 2022 – Jan 2023 (rapid test period)**	SC	0.4875 (p = 0.128272)	0.9071 (p = 0.000116)	0.6123 (p = 0.045226)
**Apr 2020 – Jan 2023 (full data)**	VT	0.6307 (p = 8.3e-05)	Insufficient data	Insufficient data
**Apr 2020 – Jan 2022 (pre-rapid test period)**	VT	Insufficient data	Insufficient data	Insufficient data
**Feb 2022 – Jan 2023 (rapid test period)**	VT	0.3524 (p = 0.287882)	-0.2982 (p = 0.373104)	-0.7811 (p = 0.004536)
**Apr 2020 – Jan 2023 (full data)**	AZ	0.7387 (p = 1e-06)	Insufficient data	Insufficient data
**Apr 2020 – Jan 2022 (pre-rapid test period)**	AZ	0.9103 (p = 0.0)	Insufficient data	Insufficient data
**Feb 2022 – Jan 2023 (rapid test period)**	AZ	0.1653 (p = 0.627195)	0.8001 (p = 0.003103)	0.1438 (p = 0.673149)
**Apr 2020 – Jan 2023 (full data)**	WI	0.7765 (p = 0.0)	Insufficient data	Insufficient data
**Apr 2020 – Jan 2022 (pre-rapid test period)**	WI	0.9215 (p = 0.0)	Insufficient data	Insufficient data
**Feb 2022 – Jan 2023 (rapid test period)**	WI	0.2774 (p = 0.408833)	0.8324 (p = 0.001475)	0.1852 (p = 0.585717)
**Apr 2020 – Jan 2023 (full data)**	AR	0.7431 (p = 1e-06)	Insufficient data	Insufficient data
**Apr 2020 – Jan 2022 (pre-rapid test period)**	AR	0.8834 (p = 0.0)	Insufficient data	Insufficient data
**Feb 2022 – Jan 2023 (rapid test period)**	AR	0.758 (p = 0.006871)	-0.0763 (p = 0.823611)	-0.1209 (p = 0.723271)
**Apr 2020 – Jan 2023 (full data)**	KS	0.5675 (p = 0.000573)	Insufficient data	Insufficient data
**Apr 2020 – Jan 2022 (pre-rapid test period)**	KS	0.7957 (p = 1e-05)	Insufficient data	Insufficient data
**Feb 2022 – Jan 2023 (rapid test period)**	KS	0.3634 (p = 0.271923)	0.7607 (p = 0.006563)	0.4189 (p = 0.19967)
**Apr 2020 – Jan 2023 (full data)**	NC	0.7245 (p = 2e-06)	Insufficient data	Insufficient data
**Apr 2020 – Jan 2022 (pre-rapid test period)**	NC	0.9219 (p = 0.0)	Insufficient data	Insufficient data
**Feb 2022 – Jan 2023 (rapid test period)**	NC	0.463 (p = 0.151521)	0.7768 (p = 0.00492)	0.353 (p = 0.286984)
**Apr 2020 – Jan 2023 (full data)**	TX	0.8047 (p = 0.0)	Insufficient data	Insufficient data
**Apr 2020 – Jan 2022 (pre-rapid test period)**	TX	0.9207 (p = 0.0)	Insufficient data	Insufficient data
**Feb 2022 – Jan 2023 (rapid test period)**	TX	0.6588 (p = 0.0275)	0.6221 (p = 0.040975)	0.2459 (p = 0.466065)
**Apr 2020 – Jan 2023 (full data)**	UT	0.7591 (p = 0.0)	Insufficient data	Insufficient data
**Apr 2020 – Jan 2022 (pre-rapid test period)**	UT	0.9447 (p = 0.0)	Insufficient data	Insufficient data
**Feb 2022 – Jan 2023 (rapid test period)**	UT	0.7162 (p = 0.01316)	0.5196 (p = 0.10138)	0.6158 (p = 0.04368)
**Apr 2020 – Jan 2023 (full data)**	WV	0.6368 (p = 6.8e-05)	Insufficient data	Insufficient data
**Apr 2020 – Jan 2022 (pre-rapid test period)**	WV	0.8918 (p = 0.0)	Insufficient data	Insufficient data
**Feb 2022 – Jan 2023 (rapid test period)**	WV	0.6785 (p = 0.021709)	0.57 (p = 0.067144)	0.2239 (p = 0.508015)
**Apr 2020 – Jan 2023 (full data)**	AL	0.8425 (p = 0.0)	Insufficient data	Insufficient data
**Apr 2020 – Jan 2022 (pre-rapid test period)**	AL	0.9297 (p = 0.0)	Insufficient data	Insufficient data

© 2024 Santillana M et al. *JAMA Network Open.*

**Feb 2022 – Jan 2023 (rapid test period)**	AL	0.7204 (p = 0.012402)	0.092 (p = 0.787884)	0.1043 (p = 0.760276)
**Apr 2020 – Jan 2023 (full data)**	LA	0.7408 (p = 1e-06)	Insufficient data	Insufficient data
**Apr 2020 – Jan 2022 (pre-rapid test period)**	LA	0.8853 (p = 0.0)	Insufficient data	Insufficient data
**Feb 2022 – Jan 2023 (rapid test period)**	LA	0.5568 (p = 0.075228)	0.2716 (p = 0.419066)	0.4731 (p = 0.14164)
**Apr 2020 – Jan 2023 (full data)**	MS	0.6914 (p = 8e-06)	Insufficient data	Insufficient data
**Apr 2020 – Jan 2022 (pre-rapid test period)**	MS	0.8956 (p = 0.0)	Insufficient data	Insufficient data
**Feb 2022 – Jan 2023 (rapid test period)**	MS	0.4599 (p = 0.15463)	0.7206 (p = 0.012353)	0.3361 (p = 0.312168)
**Apr 2020 – Jan 2023 (full data)**	MO	0.6598 (p = 3e-05)	Insufficient data	Insufficient data
**Apr 2020 – Jan 2022 (pre-rapid test period)**	MO	0.8221 (p = 3e-06)	Insufficient data	Insufficient data
**Feb 2022 – Jan 2023 (rapid test period)**	MO	0.4917 (p = 0.124541)	0.428 (p = 0.189073)	0.1276 (p = 0.708419)
**Apr 2020 – Jan 2023 (full data)**	NH	0.7256 (p = 2e-06)	Insufficient data	Insufficient data
**Apr 2020 – Jan 2022 (pre-rapid test period)**	NH	0.9665 (p = 0.0)	Insufficient data	Insufficient data
**Feb 2022 – Jan 2023 (rapid test period)**	NH	-0.1571 (p = 0.644551)	-0.2385 (p = 0.479942)	-0.3909 (p = 0.234609)
**Apr 2020 – Jan 2023 (full data)**	OH	0.8445 (p = 0.0)	Insufficient data	Insufficient data
**Apr 2020 – Jan 2022 (pre-rapid test period)**	OH	0.9667 (p = 0.0)	Insufficient data	Insufficient data
**Feb 2022 – Jan 2023 (rapid test period)**	OH	0.7766 (p = 0.004939)	0.6322 (p = 0.036889)	0.4867 (p = 0.128978)
**Apr 2020 – Jan 2023 (full data)**	HI	0.9004 (p = 0.0)	Insufficient data	Insufficient data
**Apr 2020 – Jan 2022 (pre-rapid test period)**	HI	0.9731 (p = 0.0)	Insufficient data	Insufficient data
**Feb 2022 – Jan 2023 (rapid test period)**	HI	0.8599 (p = 0.000687)	0.348 (p = 0.294342)	0.4845 (p = 0.131016)
**Apr 2020 – Jan 2023 (full data)**	OK	0.6894 (p = 9e-06)	Insufficient data	Insufficient data
**Apr 2020 – Jan 2022 (pre-rapid test period)**	OK	0.8422 (p = 1e-06)	Insufficient data	Insufficient data
**Feb 2022 – Jan 2023 (rapid test period)**	OK	0.7484 (p = 0.008058)	0.3519 (p = 0.288587)	0.5257 (p = 0.096757)
**Apr 2020 – Jan 2023 (full data)**	WY	0.827 (p = 0.0)	Insufficient data	Insufficient data
**Apr 2020 – Jan 2022 (pre-rapid test period)**	WY	Insufficient data	Insufficient data	Insufficient data
**Feb 2022 – Jan 2023 (rapid test period)**	WY	0.5097 (p = 0.109263)	Insufficient data	Insufficient data
**Apr 2020 – Jan 2023 (full data)**	DC	0.6422 (p = 5.6e-05)	Insufficient data	Insufficient data
**Apr 2020 – Jan 2022 (pre-rapid test period)**	DC	0.9274 (p = 0.0)	Insufficient data	Insufficient data
**Feb 2022 – Jan 2023 (rapid test period)**	DC	0.6751 (p = 0.022638)	0.3331 (p = 0.316836)	-0.1383 (p = 0.685046)
**Apr 2020 – Jan 2023 (full data)**	GA	0.7048 (p = 5e-06)	Insufficient data	Insufficient data
**Apr 2020 – Jan 2022 (pre-rapid test period)**	GA	0.8661 (p = 0.0)	Insufficient data	Insufficient data
**Feb 2022 – Jan 2023 (rapid test period)**	GA	0.3228 (p = 0.332991)	Insufficient data	Insufficient data
**Apr 2020 – Jan 2023 (full data)**	NM	0.6437 (p = 5.3e-05)	Insufficient data	Insufficient data
**Apr 2020 – Jan 2022 (pre-rapid test period)**	NM	0.8884 (p = 0.0)	Insufficient data	Insufficient data
**Feb 2022 – Jan 2023 (rapid test period)**	NM	0.2396 (p = 0.477896)	Insufficient data	Insufficient data
**Apr 2020 – Jan 2023 (full data)**	MI	0.6783 (p = 1.4e-05)	Insufficient data	Insufficient data
**Apr 2020 – Jan 2022 (pre-rapid test period)**	MI	0.85 (p = 1e-06)	Insufficient data	Insufficient data
**Feb 2022 – Jan 2023 (rapid test period)**	MI	0.2704 (p = 0.421342)	0.0894 (p = 0.793821)	-0.1524 (p = 0.654552)

© 2024 Santillana M et al. *JAMA Network Open.*

**Apr 2020 – Jan 2023 (full data)**	SD	0.7891 (p = 0.0)	Insufficient data	Insufficient data
**Apr 2020 – Jan 2022 (pre-rapid test period)**	SD	0.8654 (p = 0.0)	Insufficient data	Insufficient data
**Feb 2022 – Jan 2023 (rapid test period)**	SD	0.6975 (p = 0.017039)	Insufficient data	Insufficient data
**Apr 2020 – Jan 2023 (full data)**	AK	0.4565 (p = 0.007577)	Insufficient data	Insufficient data
**Apr 2020 – Jan 2022 (pre-rapid test period)**	AK	0.517 (p = 0.013735)	Insufficient data	Insufficient data
**Feb 2022 – Jan 2023 (rapid test period)**	AK	0.5216 (p = 0.099878)	Insufficient data	Insufficient data
**Apr 2020 – Jan 2023 (full data)**	ND	0.8262 (p = 0.0)	Insufficient data	Insufficient data
**Apr 2020 – Jan 2022 (pre-rapid test period)**	ND	0.8735 (p = 0.0)	Insufficient data	Insufficient data
**Feb 2022 – Jan 2023 (rapid test period)**	ND	0.6444 (p = 0.032354)	Insufficient data	Insufficient data

© 2024 Santillana M et al. *JAMA Network Open.*

eTable 3. Monthly observed COVID-19 cases estimated from survey data for the multiple survey deployments

Month	Corresponding contemporaneous wave	Fielding date	Sample Size	CSP Confirmed COVID cases estimate (% population (SE))	Conf int (±)	Official new cases (% population)	Monthly observed wastewater virus copies (SARS-CoV-2 copies / mL of sewage)
1/1/20	W5	06/12/20 - 06/28/20	22905	0.17 (0.04)	0.08	0	0
2/1/20	W5	06/12/20 - 06/28/20	22905	0.4 (0.06)	0.12	0	4.351
3/1/20	W5	06/12/20 - 06/28/20	22905	0.53 (0.07)	0.14	0.057	5964.138
4/1/20	W5	06/12/20 - 06/28/20	22905	0.44 (0.06)	0.11	0.267	16286.723
5/1/20	W5	06/12/20 - 06/28/20	22905	0.35 (0.05)	0.1	0.218	3403.032
6/1/20	W9	08/07/20 - 08/26/20	21496	0.47 (0.06)	0.13	0.257	3350.295
7/1/20	W9	08/07/20 - 08/26/20	21496	0.61 (0.08)	0.15	0.578	5485.554
8/1/20	W10	09/04/20 - 09/30/20	23050	0.43 (0.05)	0.09	0.444	3713.291
9/1/20	W11	10/02/20 - 10/23/20	19570	0.43 (0.06)	0.11	0.367	2158.504
10/1/20	W13	11/03/20 - 11/30/20	26642	0.76 (0.06)	0.12	0.586	5015.679
11/1/20	W14	12/16/20 - 01/10/21	26113	1.67 (0.11)	0.21	1.327	15875.933
12/1/20	W16	02/05/21 - 02/28/21	23348	1.84 (0.13)	0.25	1.931	22093.742
1/1/21	W16	02/05/21 - 02/28/21	23348	1.33 (0.10)	0.2	1.867	17244.19
2/1/21	W17	04/01/21 - 05/03/21	23718	0.69 (0.07)	0.14	0.724	5824.943
3/1/21	W17	04/01/21 - 05/03/21	23718	0.52 (0.07)	0.13	0.559	5721.44
4/1/21	W18	06/09/21 - 07/15/21	22275	0.45 (0.06)	0.11	0.567	4763.132
5/1/21	W18	06/09/21 - 07/15/21	22275	0.34 (0.05)	0.1	0.276	1804.246
6/1/21	W19	08/26/21 - 09/27/21	23938	0.26 (0.04)	0.08	0.109	1792.336
7/1/21	W19	08/26/21 - 09/27/21	23938	0.44 (0.05)	0.1	0.415	6299.917
8/1/21	W19	08/26/21 - 09/27/21	23938	1.07 (0.08)	0.16	1.295	17335.669
9/1/21	W20	11/03/21 - 12/02/21	24623	1.35 (0.09)	0.18	1.247	16999.981
10/1/21	W20	11/03/21 - 12/02/21	24623	0.96 (0.07)	0.14	0.746	13916.669
11/1/21	W21	12/22/21 - 01/24/22	25358	1.25 (0.09)	0.17	0.779	23814.523
12/1/21	W22	03/02/22 - 04/09/22	23376	3.3 (0.15)	0.29	1.858	80914.958
1/1/22	W22	03/02/22 - 04/09/22	23376	5.95 (0.19)	0.38	6.114	75543.053
2/1/22	W22	03/02/22 - 04/09/22	23376	1.82 (0.11)	0.21	1.191	7985.319
3/1/22	W23	06/08/22 - 07/05/22	24625	1.12 (0.09)	0.17	0.32	4155.093
4/1/22	W23	06/08/22 - 07/05/22	24625	1.16 (0.08)	0.15	0.378	10418.822
5/1/22	W23	06/08/22 - 07/05/22	24625	2.04 (0.11)	0.21	0.868	21505.548
6/1/22	W24	08/11/22 - 09/11/22	26557	2.46 (0.12)	0.23	1.002	24605.985
7/1/22	W24	08/11/22 - 09/11/22	26557	3.24 (0.13)	0.26	1.098	32976.992
8/1/22	W25	10/06/22 - 11/09/22	25964	2.73 (0.12)	0.23	0.97	23397.223
9/1/22	W25	10/06/22 - 11/09/22	25964	2.26 (0.11)	0.22	0.545	21324.956
10/1/22	W26	12/22/22 - 01/17/23	24957	2.24 (0.11)	0.22	0.328	18449.675
11/1/22	W26	12/22/22 - 01/17/23	24957	2.09 (0.11)	0.21	0.386	20673.142
12/1/22	W26	12/22/22 - 01/17/23	24957	2.5 (0.12)	0.24	0.586	32559.616

© 2024 Santillana M et al. *JAMA Network Open.*

eTable 4. The differences in number of cases recorded during the period after rapid tests were deployed on ground (Feb'22 to Dec'22) between Official data source (New York Times) and Covid States survey and prediction obtained by training a linear regression using Covid states.

	state	CSP to Official difference in cases	Prediction to Official difference in cases
**0**	AL	526990.2407286850	339167.7661272380
**1**	AK	130722.77686534600	70925.83735815010
**2**	AZ	1163460.812393650	939389.6976879230
**3**	AR	331598.5153564970	310298.39594262800
**4**	CA	6333548.762619290	3956958.538897410
**5**	CO	709087.9785993950	649805.9267643760
**6**	CT	582022.3111240730	421152.6409606460
**7**	DE	114836.66901829700	94628.34574050710
**8**	DC	146331.68562350000	123482.81987125900
**9**	FL	2649599.595303260	3024054.5740217500
**10**	GA	1281834.23527784	847755.7283917550
**11**	HI	254350.86896302200	143971.60473489300
**12**	ID	373036.4852108260	133555.09081505300
**13**	IL	2160972.25154087	1596224.6016380500
**14**	IN	1046717.0746397500	819152.6839750710
**15**	IA	498271.9393265960	283134.3278201050
**16**	KS	435905.8917539130	469177.11598783900
**17**	KY	669161.2321769830	527628.9195764120
**18**	LA	528896.3040216120	467074.23265232600
**19**	ME	330705.8162441610	70882.51071924790
**20**	MD	990107.8256367590	437804.18152309500
**21**	MA	1879799.094044760	1556633.1033855300
**22**	MI	1494072.052463090	1234583.1526418400
**23**	MN	987538.7609220750	852918.7782339500

© 2024 Santillana M et al. *JAMA Network Open.*

**24**	MS	390256.87324185500	349246.0755054090
**25**	MO	661258.7671207210	499102.54315186600
**26**	MT	185205.11935427600	95067.28512833800
**27**	NE	275152.0241479130	215930.52934384500
**28**	NV	394434.1017132090	307211.98362026000
**29**	NH	240680.32445473500	182951.9719613760
**30**	NJ	1126418.2797025100	916286.2946430220
**31**	NM	362858.2912353530	293250.7174076290
**32**	NY	3370318.1461734700	3113796.2989901000
**33**	NC	1743489.3303337700	1705378.2399059200
**34**	ND	81468.70089653900	42395.14151424800
**35**	OH	1580612.9193829400	1101912.833284720
**36**	OK	773118.397172827	557320.7748654880
**37**	OR	970239.3458168210	855289.4586586090
**38**	PA	2273632.831496540	1519336.359492450
**39**	RI	156100.9546575740	173908.50290833200
**40**	SC	611823.517653686	792150.9439946130
**41**	SD	133829.881385874	77775.3258424032
**42**	TN	717906.5104187630	552970.7700769660
**43**	TX	3700810.7438716500	2104511.527014770
**44**	UT	610019.1389372450	473928.36431214400
**45**	VT	220597.6522698980	163321.01305611200
**46**	VA	1474063.6320805200	932795.0678966490
**47**	WA	1440193.745535710	1159384.3790950000
**48**	WV	405224.1315189560	375163.860850622
**49**	WI	902308.1077957830	719750.8848672150
**50**	WY	59558.02379955020	16852.325126042100

© 2024 Santillana M et al. *JAMA Network Open.*

eTable 5. Number of state-level unreported infection per 100,000 individuals as calculated from survey data

State	Unreported cases pre-rapid test (per 100,000)	Unreported cases post-rapid test (per 100,000)	State's population
VT	3,528	34,171	645,570
MA	3,080	26,913	6,984,723
ME	6,556	24,100	1,372,247
OR	3,916	22,850	4,246,155
WV	576	22,728	1,782,959
DC	5,193	21,715	670,050
ID	11,914	19,625	1,900,923
OK	5,542	19,393	3,986,639
WA	4,022	18,662	7,738,692
UT	5,211	18,280	3,337,975
AK	7,569	18,065	732,673
PA	4,176	17,538	12,964,056
HI	5,420	17,369	1,441,553
NH	3,140	17,328	1,388,992
MN	1,383	17,300	5,707,390
NM	2,917	17,149	2,115,877
VA	6,026	17,128	8,642,274
IL	2,707	17,072	12,671,469
NY	1,087	17,019	19,835,913
MT	9,134	16,721	1,104,271
NC	-2,193	16,529	10,551,162
MD	8,233	16,418	6,165,129
CA	7,341	16,193	39,237,836
CT	4,108	16,116	3,605,597
AZ	6,196	15,990	7,276,316
IA	6,393	15,605	3,193,079
IN	5,401	15,322	6,805,985
WI	3,603	15,304	5,895,908
SD	10,922	14,947	895,376
MI	1,402	14,832	10,050,811
KS	-895	14,828	2,934,582
KY	2,712	14,519	4,509,394
RI	-3,438	14,248	1,095,610

© 2024 Santillana M et al. *JAMA Network Open.*

CO	-574	14,132	5,812,069
NE	4,507	14,090	1,963,692
OH	5,464	13,418	11,780,017
MS	1,947	13,229	2,949,965
NV	1,266	12,546	3,143,991
TX	9,382	12,243	29,527,941
NJ	1,465	12,155	9,267,130
FL	-1,702	11,848	21,781,128
GA	5,267	11,677	10,799,566
DE	1,641	11,445	1,003,384
LA	454	11,438	4,624,047
SC	-2,174	11,211	5,190,705
AR	1,714	10,959	3,025,891
ND	10,263	10,729	774,948
MO	5,071	10,713	6,168,187
AL	6,154	10,550	5,039,877
TN	2,718	10,345	6,975,218
WY	12,667	10,290	578,803

© 2024 Santillana M et al. *JAMA Network Open.*

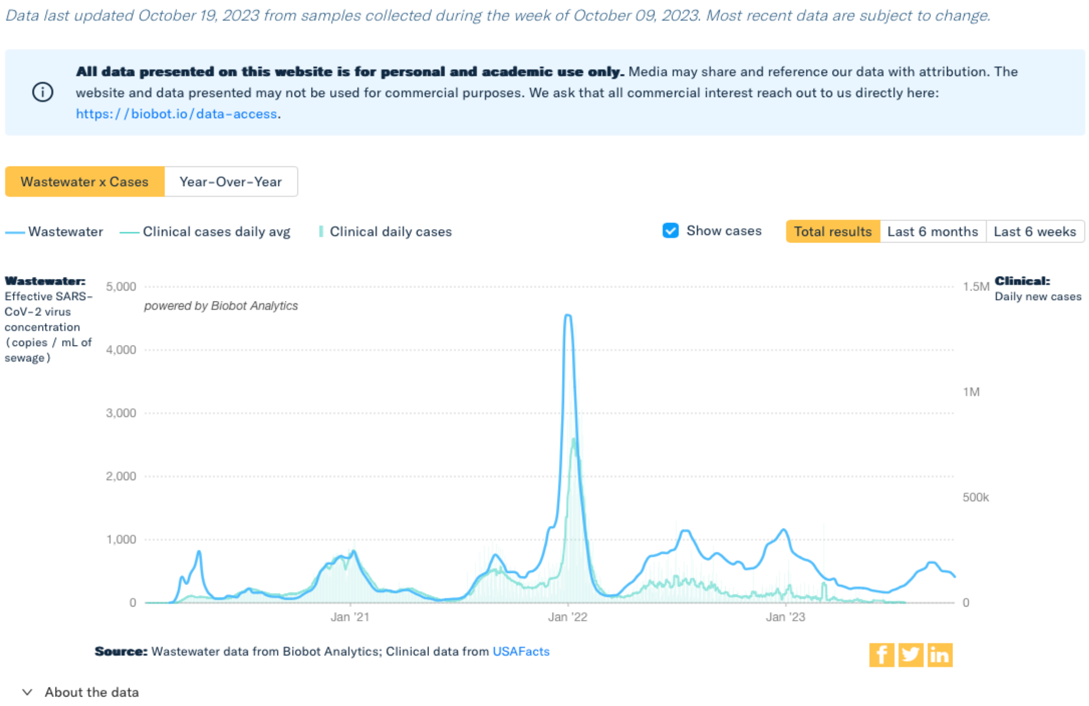

eFigure 9. Case estimation performed by BioBot using viral concentration in wastewater.

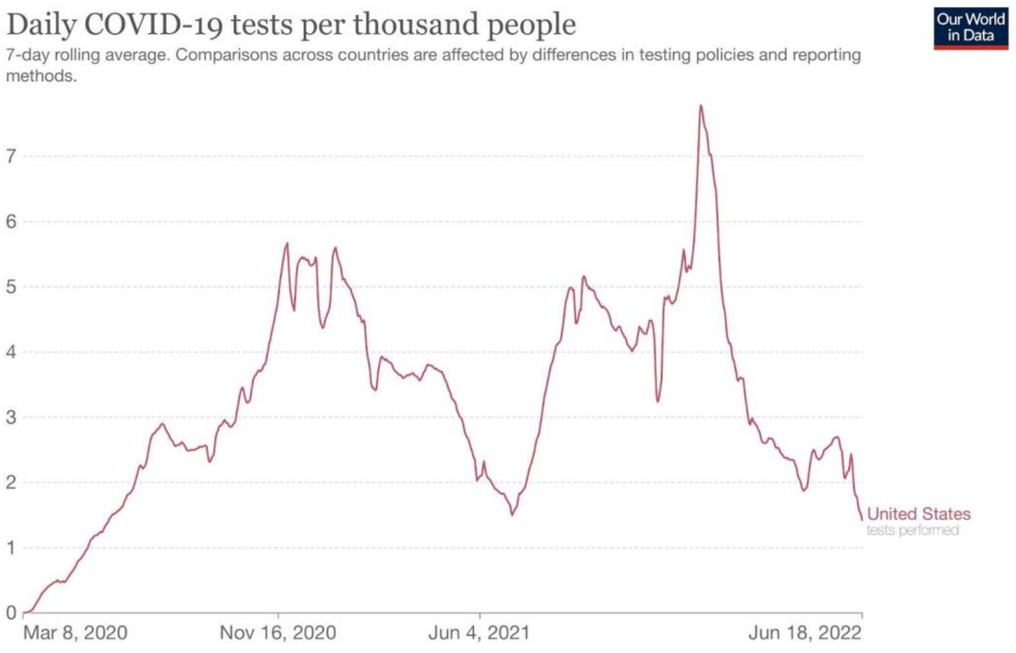

eFigure 10. Daily COVID-19 tests administered in the United States per thousand people. The data and the figure are from Our World In Data.

Infection Curves Sensitivity Analysis (Excluding repeat respondents).

In order to assess the extent to which our results would vary when including repeat respondents (and potential repeat infections in our analysis), we conducted multiple experiments where we estimated the number of infections in a given point in time, by only including repeat respondents only once (chosen randomly) in our longitudinal analysis. The following plots show that the resulting infections curves estimated from these experiments are very similar to the one that was obtained from including repeat respondents.

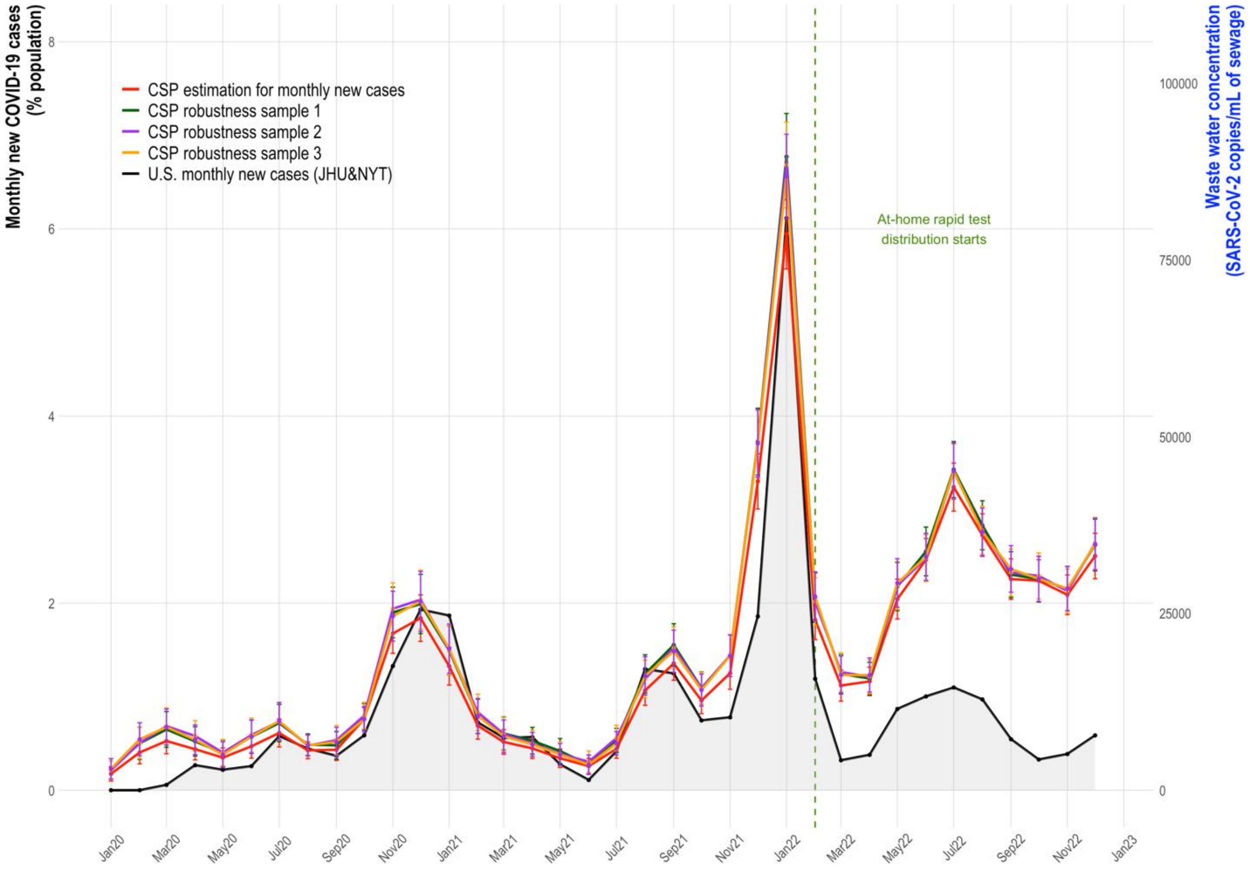

eFigure 11. Sensitivity analysis of infection curves obtained by only including repeat respondents only once (chosen randomly) in our longitudinal analysis.

© 2024 Santillana M et al. *JAMA Network Open.*
