# Supplementary material for: Tracking COVID-19 Infections Using Survey Data on Rapid At-Home Tests
Source: JAMA Netw Open. 2024 Sep 30;7(9):e2435442. doi: 10.1001/jamanetworkopen.2024.35442 (PMC11443354; doi:10.1001/jamanetworkopen.2024.35442)
Supplement: Supplement 2. — Data Sharing Statement [file jamanetwopen-e2435442-s002.pdf]

Data Sharing Statement

Santillana. Tracking COVID-19 Infections Using Survey Data on Rapid At-Home Tests. *JAMA Netw Open*. Published September 30, 2024. doi:10.1001/jamanetworkopen.2024.35442

Data
Data available: Yes
Data types: Deidentified participant data
How to access data: katya.ognyanova@rutgers.edu
When available: With publication

Supporting Documents
Document types: None

Additional Information
Who can access the data: researchers whose proposed use of the data has been approved
Types of analyses: Research
Mechanisms of data availability: with a signed data access agreement
